# Implantation of Short-Term and Long-Term Right Ventricular Assist Devices

**DOI:** 10.21470/1678-9741-2017-0021

**Published:** 2017

**Authors:** Christiano Castello Branco Caldeira, Regimar Carla Machado, Debora Castello Branco Caldeira

**Affiliations:** 1 Tampa General Hospital, FACT Surgery Division Cardiothoracic Surgery, University of South Florida, Tampa, FL, USA.; 2 Universidade Federal de São Carlos (UFSCar), São Carlos, SP, Brazil.; 3 Faculdade de Medicina de Barbacena (FAME), Barbacena, MG, Brazil.

**Keywords:** Heart Failure, Right Ventricular Failure, Ventricular Assist Devices

## Abstract

The last decade has seen considerable growth in the use of left ventricular assist devices (LVAD), in end-phase heart failure treatment. The indications, contraindications and implantation techniques are well-defined. However, information about mechanical support for right ventricular failure is lacking. The aim of this communication is to present alternative techniques for implantation of short- and longterm right ventricular assist devices. Implanting the device in the right atrium has certain advantages when compared with the right ventricle. It is an easier surgical technique that preserves the tricuspid valve and it can potentially reduce the risk of pump thrombosis.

**Table:** 

Abbreviations, acronyms & symbols	
CO	= Cardiac output
CVP	= Central venous pressure
FDA	= Food and Drug Administration
LVAD	= Left ventricular assist device
NYHA	= New York Heart Association
PA	= Pulmonary artery
PCWP	= Pulmonary capillary wedge pressure
PVR	= Pulmonary vascular resistance
RA	= Right atrium
RV	= Right ventricle
RVAD	= Right ventricular assist device
VAD	= Ventricular assist device

## INTRODUCTION

Refractory right ventricular failure has been defined as needing inotropic or vasodilator support, or a right ventricular assist device (RVAD). Right ventricular failure following the implantation of ventricular assist devices (VAD) has been shown to be a strong predictor of increased postoperative morbidity and mortality^[1]^. It is believed that low intraventricular pressure caused by left ventricular assist devices (LVAD) can lead to a shift in the position of the interventricular septum (bulging to the left). Consequently, the right ventricle (RV) loses its triangular shape and ability to contract against the intraventricular septum, causing increased right ventricular volume and worsening of tricuspid regurgitation^[1,2]^.

The last decade has seen considerable growth in the use of LVAD in end-phase heart failure treatment. The indications, contraindications and implantation techniques are well-defined. However, the same cannot be said about mechanical support for right ventricular failure. Mechanical support for RV defects has been performed with paracorporeal pumps. Some studies describe the use of intracorporeal LVAD to support failing RV. However, there is lack of information regarding optimal cannulation site, pump rotor speed, flow, waveforms, and anticoagulation.

The aim of this communication is to present alternative techniques for implanting short- and long-term RVAD.

## CASE ILLUSTRATION

### Case #1

A 52-year-old woman with a history of end-stage heart failure, secondary to ischemic cardiomyopathy, and undergoing optimized medical therapy presented New York Heart Association (NYHA) class III symptoms associated with signs of biventricular dysfunction. The transthoracic echocardiogram demonstrated global hypokinesis and an estimated ejection fraction of 15%, also severe mitral valve regurgitation and right ventricular dilation. Right cardiac catheterization confirmed low cardiac output (CO: 2.8 L/min - index 1.5 L/min/m^2^), elevated central venous pressure (CVP: 16 mmHg), systolic pulmonary hypertension (PA: 72 mmHg), pulmonary vascular resistance (PVR: 629 dynes/sec/cm^5^), and the pulmonary capillary wedge pressure (PCWP: 20 mmHg).

The patient was started on intravenous inotropic therapy (milrinone 0.5 mcg/kg/min), a systemic vasoconstrictor (vasopressin 0.02 mcg/kg/min) and a diuretic (furosemide 10 mg/h). After clinical improvement and complete preoperative evaluation, the patient was presented to our multidisciplinary heart failure group for consideration of VAD implantation.

The planned operation consisted of LVAD implantation (HeartWare), tricuspid valve repair, and percutaneous implantation of temporary right ventricular mechanical support (Impella RP). After two weeks with biventricular support, with an LVAD flow of 5.2 L/min and an RVAD flow of 4.8 L/min, the echocardiogram did not demonstrate an improved right ventricular function, despite moderate doses of inotropes. The patient was returned to the operating room for the removal of the temporary RVAD support and implantation of an intracorporeal RVAD (HeartWare) for long-term support.

During a short extracorporeal circulation period, HeartWare was implanted in the right pleural space with the inflow cannula tunneled through the pericardium and lateral wall of the right atrium (RA). Several felt rings were attached to the sewing ring to adjust the depth of the inflow cannula in the right atrial cavity. An outflow graft with no flow restriction was anastomosed to the main pulmonary artery.

The devices maintained excellent flow, with the RVAD operating at 5.2 L/min at 2400 rpm and the LVAD at 5.6 L/min. After satisfactory evolution of clinical, laboratory and radiological parameters, the patient was weaned off the mechanical ventilator and was discharged to home. Patient underwent transplantation seven months after VAD implantation.

### Case #2

A 62-year-old woman with history of dilated cardiomyopathy, on the list for an orthotopic heart transplant. While waiting for the transplant and receiving intravenous inotropic medication, she suffered severe hemodynamic decompensation.

The echocardiogram demonstrated low left ventricular ejection fraction and moderate right ventricular dysfunction. Right cardiac catheterization showed systolic pulmonary hypertension (PA: 4 mmHg), elevated CVP (12 mmHg), low CO (1.6 L/min/m^2^), PVR (509 dynes/sec/cm^5^), and the PCWP (18 mmHg).

The patient underwent implantation of a LVAD (HeartMate II). The immediate postoperative period was marked by low pulsatile flow of the left ventricular device associated with low pulsatility index, high CVP, systemic arterial hypotension, and low CO. The echocardiogram showed satisfactory cannula position, enlarged RA and RV, and severe tricuspid regurgitation.

After two days of RV support with high doses of inotropic medication, financial authorization was granted for the implantation of an RVAD. During a short period of extracorporeal circulation, the defibrillator leads were removed and a HeartWare VAD was implanted with an inflow cannula through the diaphragmatic surface of the RV. The inflow cannula was adjusted to a suitable depth. The external outflow graft, with 50% reduction in diameter, was anastomosed to the main pulmonary artery.

Excellent flow was obtained with the HeartWare and HeartMate II pumps operating at 2400 rpm and 8200 rpm, respectively.

On postoperative day 5, the patient presented an abrupt decrease in left ventricle support flow associated with elevated CVP, reduced CO, and elevation in HeartWare RVAD pump power. Laboratory exams were compatible with hemolysis.

During the emergency surgery, a thrombus was confirmed in the RVAD. The tricuspid valve leaflets were partially obstructing the RVAD inflow cannula, predisposing to stagnated flow and thrombosis.

After tricuspid valve resection, a new VAD was implanted (HeartWare) with no restriction in the outflow graft. With the new device operating at 2200 rpm, excellent biventricular flow was obtained. The HeartWare RVAD was removed and another HeartWare was implanted.

The patient recovered well and she was eventually discharged to home. Six months after the implantation, the patient underwent a successful heart transplantation.

## DISCUSSION

In general, patients with biventricular assist devices present lower survival rates in comparison with those with single LVAD (53% *vs.* 80%). The inferior survival rates of patients with biventricular support can be explained by patient selection and/ or increased incidence of associated comorbidities. However, considering the lack of best practices related to technique, VAD adjustments, anticoagulation, and postoperative management of patients implanted with intracorporeal biventricular support devices, it is possible that many of these complications could be prevented^[1,3]^.

In our service, temporary ventricle support is performed by inserting pulmonary artery cannulation through the right jugular vein using a flexible wire-reinforced single-stage 17 Fr cannula. Venous drainage is performed using a multiple-stage 25 Fr cannula introduced through the right femoral artery into the RA. Flow can reach up to 5 L/min by connecting the cannula to a centrifugal pump (Centrimag or Sorin). A similar technique has been described^[4]^ with arterial pulmonary cannulation through the femoral vein.

Recently, the Impella RP was approved by the Food and Drug Administration (FDA) for temporary percutaneous RV support. This device is implanted through the right femoral vein and is fluoroscopically guided into the pulmonary trunk. Its maximum motor speed generates flows up to 5 L/min. The authors' experience with the Impella RP has been limited, but it has yielded very good outcomes.

However, if the RV function is not recovered after some weeks of temporary support, the implantation of long-term intracorporeal mechanical circulatory support is indicated^[3,4]^.

Currently, there are no long-term intracorporeal devices specifically designed to provide RV support.

The implantation of a left mechanical circulatory support device to assist a failing RV has been performed successfully at some centers. Most of the implantations are performed using personal technical modifications required to adjust to physiological and anatomical differences in right-side circulation. HeartWare is the most commonly used device for RV support^[3]^.

The surgical technique consists of implanting the HeartWare inflow cannula through the free wall of the RA or the diaphragmatic surface of the RV. Since the length of the inflow cannula is designed for the left ventricle, anatomical differences in the right chambers require changes to the surgical technique to prevent inflow cannula obstruction. The depth of the inflow cannula must be adjusted by adding hand-made felt rings underneath the anchoring ring.

Implantations in the RA tend to be easier, because resecting the tricuspid valve is not necessary. The pump is positioned in the right pleural space with the inflow cannula tunneling through the pericardium and the wall of the RA. Positioning the pump in the pleural space prevents compressing the RA, which occurs when the pump is placed on the pericardial sac.

Implantation of mechanical circulatory assist devices in the RV requires caution regarding resection of the tricuspid valve and subvalvular apparatus, and placement of any stimulation or defibrillator leads. Otherwise, the risk of inflow obstruction, thrombus ingestion and pump thrombosis increases.

The outflow graft is anastomosed to the pulmonary trunk. However, due to decreased vascular resistance on the right side of the heart, the mechanical circulatory support device can produce excessive flow into the pulmonary system. To avoid pulmonary congestion, the HeartWare device must be adjusted to a slower speed (rpm). Another option is to restrict the outflow graft, reducing its luminal diameter. This can be done by suturing a smaller graft end to the HeartWare graft or attaching vascular clips, as described in a previous study^[5]^. Little is known about the extent of graft restriction or the ideal speed of HeartWare device to overcome decreased vascular resistance. Both techniques can lead to increased thrombogenicity in comparison with left-side implants, especially when implants are placed on the diaphragmatic surface of the RV^[5,6]^.

The authors' first experiences with implantation on the RV diaphragmatic surface resulted in pump thrombosis in two out of five patients. A similar experience was described in a recently published study, in which 13 patients with dilated cardiomyopathy and severe biventricular failure were implanted with HeartWare devices. Mechanical circulatory support device thrombosis took place in three out of six patients with implants in the RV, in contrast with only one out of seven patients with devices implanted in the RA.

The cases described in the present study illustrate the complexity of clinical decisions when treating right heart failure. Given the absence of circulatory assist devices developed and approved for right-side support, when faced with patients with biventricular dysfunction, surgeons tend to adopt alternative techniques whose effectiveness has not yet been proven. Modifications to LVAD and technical alterations to adapt them to the anatomical and physiological characteristics of right-side circulation can lead to serious consequences for the success of clinical treatment.

## CONCLUSION

Implantation of devices in the RA could present advantages when compared to implantation in the RV. The required surgical technique is easier, it preserves the tricuspid valve and can potentially reduce the risk of pump thrombosis. Little is known about the best pump speed and afterload reduction to establish adequate flow for correct circulation.

Companies involved in developing and manufacturing mechanical circulatory support devices should be encouraged to invest in the development of systems specifically designed to provide long-term support to the RV.

This communication underscores the benefits of this life-saving therapy, when clinically indicated.

**Table:** 

Authors' roles & responsibilities	
CCBC	Original idea, technical support and writing of the paper; final approval of the version to be published
RCM	Study design and writing of the paper; final approval of the version to be published
DCBC	Writing and formatting of the text; final approval of the version to be published
